# Evaluation of Serum Vascular Endothelial Growth Factor (VEGF) and Placental Growth Factor (PlGF) Levels in Unexplained Early Pregnancy Loss: A Case-Control Study

**DOI:** 10.7759/cureus.111535

**Published:** 2026-06-26

**Authors:** Mahantesh BB, Nilima Dongre, Ashalata Mallapur

**Affiliations:** 1 Department of Biochemistry, Bharatiya Lingayat Development Educational Association (Deemed to be University), Vijayapura, IND; 2 Department of Obstetrics and Gynaecology, S Nijalingappa Medical College and H.S.K. Hospital and Research Centre, Bagalkot, IND

**Keywords:** angiogenesis, biomarkers, early pregnancy loss, placental growth factor, vascular endothelial growth factor

## Abstract

Background: Early pregnancy loss, defined as spontaneous termination of pregnancy before 20 weeks of gestation or fetal weight below 500 g, remains a common obstetric complication. While several etiological factors, such as genetic abnormalities and uterine defects, are established, nearly half of the cases remain unexplained, necessitating exploration of underlying biochemical mechanisms.

Objective: To evaluate and compare serum levels of vascular endothelial growth factor (VEGF) and placental growth factor (PlGF) in women with early pregnancy loss and healthy pregnant controls of similar gestational age and to assess the association and diagnostic performance of these angiogenic markers.

Methods: A hospital-based case-control study was conducted involving 60 pregnant women aged 19-45 years with pregnancies of less than 20 weeks' gestation, including 30 cases of early pregnancy loss and 30 healthy pregnant controls with viable intrauterine pregnancies of comparable gestational age. Participants were recruited from the Department of Obstetrics and Gynecology of HSK Hospital, Bagalkot, and biochemical analysis was performed at Bharatiya Lingayat Development Educational Association (Deemed to be University) (BLDE [DU]), Vijayapura. Serum VEGF and PlGF levels were measured using enzyme-linked immunosorbent assay (ELISA). Statistical analysis was performed using IBM Corp. Released 2014. IBM SPSS Statistics for Windows, Version 20. Armonk, NY: IBM Corp., applying chi-square and independent t-tests, with results expressed as mean ± standard deviation (SD). Receiver operating characteristic (ROC) curve analysis was performed to assess diagnostic performance and derive optimal cut-off values.

Results: Serum VEGF and PlGF levels were significantly reduced in cases compared to controls (p < 0.05), indicating impaired angiogenesis in early pregnancy loss. ROC analysis showed adequate diagnostic performance for VEGF and excellent diagnostic performance for PlGF in differentiating cases from controls within this case-control sample.

Conclusion: Reduced serum VEGF and PlGF levels are associated with early pregnancy loss and may serve as adjunctive diagnostic biomarkers for identifying altered angiogenic profiles in affected women after validation in larger prospective studies.

## Introduction

Early pregnancy loss, or miscarriage, is defined as spontaneous pregnancy loss before 20 weeks of gestation and is one of the most common complications of early gestation [1]. It is predicted to influence a significant percentage of women across the world, with recurrent pregnancy loss representing a major clinical and psychological burden [2]. Epidemiological trends show that the risk of pregnancy loss depends on demographic and environmental factors, reflecting its multifactorial etiology [3]. Recurrent etiologies comprise chromosomal abnormalities, endocrine dysfunction, uterine abnormalities, thrombophilic disorders, and immunological causes, but in almost half of the cases, the etiological cause remains unidentified, stressing the necessity to enhance knowledge on pathogenic mechanisms [2].

Successful early pregnancy depends on appropriate implantation and placentation, for which vascular development at the maternal-fetal interface is critical. The placenta has a central role in facilitating the exchange of nutrients and oxygen needed for the development of a fetus, and its functional integrity is largely dependent on efficient angiogenesis [4]. Disturbed placental vascular development has been reported to cause several pregnancy complications such as miscarriage, intrauterine growth restriction, and preeclampsia [5]. Angiogenesis is regulated by a dynamic balance between growth factors and signaling mechanisms, which control endothelial cell proliferation, migration, and vascular remodeling, all of which are vital in the maintenance of early pregnancy [6].

Among the most important angiogenic regulators are vascular endothelial growth factor (VEGF) and placental growth factor (PlGF), both of which belong to the VEGF family of glycoproteins. These factors mediate endothelial cell function, increase vascular permeability, and promote new blood vessel formation [7,8]. VEGF has received considerable attention because of its role in physiological and pathological angiogenesis, acting through specific receptors to regulate vascular homeostasis [8]. PlGF is a VEGF homolog that is mainly expressed in the placenta and is considered important in trophoblast invasion and placental vascularization [9]. Altered expression or activity of these angiogenic factors may impair placental development and contribute to adverse pregnancy outcomes.

There is recent evidence that dysregulation of angiogenic signaling pathways could be a major cause of early pregnancy loss. Altered expression of VEGF, PlGF, and other growth factors has been reported in women with miscarriage or recurrent pregnancy loss [10]. The impaired trophoblast invasion and malfunctioning vascular remodeling, in most cases, related to inflammatory and endoplasmic reticulum stress responses, contribute to the placental insufficiency even further [11]. Moreover, oxidative stress was also found to impair the functions of trophoblasts by transducing trophoblast function via hypoxia-inducible factor-1 alpha (HIF-1α) and VEGF signals, resulting in impaired angiogenesis [12]. These results highlight the value of angiogenic balance in a viable pregnancy.

Still, the communication of pro-angiogenic activity (VEGF) and anti-angiogenic regulators (soluble fms-like tyrosine kinase-1, sFlt-1) further affects the vascular activity in the course of pregnancy [13]. Any disequilibrium in this strictly controlled mechanism may result in dysfunction and placental anomalies of the endothelium. Another potential genetic source of angiogenic dysregulation has also been shown in genetic defects in the VEGF gene, potentially elevating the probability of recurrent pregnancy loss, which could have a hereditary element [14]. Moreover, the low serum VEGF concentration and its receptors have also been noted in women who experience recurrent unexplained spontaneous abortion, which further supports the possibility of these biomarkers in the pathogenesis of the disease [15].

Although molecular pathways that cause pregnancy loss are better understood, reliable circulating biomarkers for assessing angiogenic dysfunction in early pregnancy loss remain limited. With angiogenesis playing a crucial role in early gestation, evaluation of circulating angiogenic factors, including VEGF and PlGF, may provide useful insight into the pathophysiology of early pregnancy loss. Assessment of their serum levels may help characterize altered angiogenic profiles in women with early pregnancy loss and support evaluation of their diagnostic performance in a case-control setting. Thus, the current study was carried out to evaluate and compare serum VEGF and PlGF levels in women with early pregnancy loss and healthy pregnant controls and to assess the association and diagnostic performance of these markers.

## Materials and methods

Study design

A hospital-based case-control study design was used to evaluate the association between serum angiogenic biomarkers and early pregnancy loss. Women diagnosed with early pregnancy loss were included as cases, whereas healthy pregnant women with viable intrauterine pregnancies of comparable gestational age were included as controls. This design allowed comparison of serum VEGF and PlGF levels between affected and unaffected pregnant women at a similar stage of gestation. The study was planned to minimize bias through clearly defined inclusion and exclusion criteria, gestational age matching, and standardized biochemical assessment, thereby improving the validity of comparisons between cases and controls.

Study setting

The study was conducted collaboratively by the Department of Biochemistry, Bharatiya Lingayat Development Educational Association (Deemed to be University), Vijayapura, and the Department of Obstetrics and Gynecology, S. Nijalingappa Medical College and H.S.K Hospital & Research Centre Hospital (HSK Hospital), Bagalkot. Participant recruitment, clinical evaluation, and diagnosis were performed in the Department of Obstetrics and Gynecology at HSK Hospital, Bagalkot, whereas biochemical analysis was performed in the Department of Biochemistry at BLDE (DU), Vijayapura, under standardized laboratory conditions. This arrangement allowed appropriate clinical enrollment and controlled laboratory assessment, thereby enhancing the reliability and reproducibility of the study findings.

Study duration

The study was conducted from February 2023 to December 2024.

Study population

Pregnant women aged 19-45 years with pregnancies of less than 20 weeks' gestation were included in the study. Participant selection was based on clinical presentation and ultrasound findings. The case group comprised women diagnosed with early pregnancy loss, whereas the control group comprised healthy pregnant women with viable intrauterine pregnancies confirmed by ultrasonography. Both groups were selected with comparable gestational ages to minimize confounding due to gestational-age-related physiological variation in biomarker levels. This uniform gestational age criterion was applied consistently to both cases and controls to maintain comparability between the groups.

Sample size and group

The number of participants included in the study was 60, divided into two equal groups: 30 cases and 30 controls. Cases comprised women diagnosed with early pregnancy loss, whereas controls comprised healthy pregnant women with viable ongoing intrauterine pregnancies of comparable gestational age. No randomization was performed because this was an observational hospital-based case-control study. Participants were assigned to the case or control group based on clinical diagnosis and ultrasound findings, rather than by random allocation. The sample size was determined based on feasibility and previous similar studies. The equal distribution between groups allowed balanced comparative analysis and helped reduce sampling imbalance, thereby improving the reliability of statistical inference. The sample size was calculated for comparison of two independent means using the following formula:

$n=\frac{2\left(Z_{\alpha / 2}+Z_\beta\right)^2 \sigma^2}{d^2}$

where n represents the required sample size per group, Zα/2 = 1.96 at a 95% confidence level, Zβ = 0.84 at 80% power, σ represents the pooled standard deviation estimated from previous similar studies, and d represents the expected difference in mean serum biomarker levels between cases and controls. Based on this calculation, the minimum required sample size was approximately 27 participants per group. Therefore, 30 participants were included in each group to improve statistical reliability and compensate for possible data loss.

Inclusion criteria

Cases included pregnant women at less than 20 weeks of gestation who presented with clinical features such as vaginal bleeding and abdominal pain and were confirmed by ultrasonography to have an absence of fetal cardiac activity. Controls included healthy pregnant women at less than 20 weeks of gestation with fetal viability confirmed by ultrasonography. Gestational age matching was performed to minimize physiological differences in biomarker levels between cases and controls. These criteria ensured that the case group represented true early pregnancy loss and that the control group represented normal ongoing pregnancies at a comparable gestational stage.

Exclusion criteria

Participants with known conditions that could affect pregnancy outcome or angiogenic marker levels were excluded to minimize confounding bias. These conditions included thyroid disorders, antiphospholipid antibody syndrome, toxoplasmosis, rubella, cytomegalovirus, herpes, and other diseases (TORCH) infections, chromosomal abnormalities, uterine anomalies, consanguineous marriage, and other systemic diseases. Early pregnancy loss was considered unexplained only after exclusion of identifiable clinical, endocrine, infectious, immunological, anatomical, genetic, and systemic causes based on clinical history, examination, available medical records, ultrasound findings, and relevant laboratory investigations. All women who presented with early pregnancy loss were clinically evaluated, and relevant investigations were reviewed or performed, as clinically indicated, to identify these exclusion criteria before enrollment. Thyroid dysfunction was assessed by thyroid function testing, antiphospholipid antibody syndrome by antiphospholipid antibody testing when clinically indicated, TORCH infection by relevant serological testing, uterine anomalies by ultrasonographic evaluation, and systemic illnesses by clinical evaluation and available medical records. Chromosomal abnormalities were excluded when documented in prior records or suspected based on available clinical information. Participants with documented thyroid dysfunction, antiphospholipid antibody positivity, TORCH infection, chromosomal abnormality, uterine anomaly, consanguinity, or systemic illness were excluded from the case group. Exclusion of these factors ensured that the observed differences in serum VEGF and PlGF levels were more likely related to unexplained early pregnancy loss rather than underlying comorbidities. This approach improved the internal validity of the study and strengthened the interpretation of biomarker associations.

Ethical considerations

Ethical approval was obtained from the institutional ethics committees of both participating institutions on January 4, 2023, before initiation of the study. All procedures were conducted in accordance with ethical principles for research involving human participants. Written informed consent was obtained from each participant after explaining the study objectives, procedures, and possible implications. Participant confidentiality was maintained throughout the study. Participation was voluntary, and participants were free to withdraw at any time without any effect on their clinical care.

Collection and processing of the samples

Each participant provided approximately 4 mL of venous blood, collected under aseptic conditions using standard phlebotomy procedures. For women in the case group, blood samples were collected before medical or surgical evacuation of the products of conception. Samples were allowed to clot at room temperature and were then centrifuged at 3000 rpm for 10 minutes to separate the serum. The obtained serum was aliquoted into labeled microcentrifuge tubes. As the biochemical analysis was performed at BLDE (DU), Vijayapura, serum samples were stored at -20°C until analysis and transported under cold-chain conditions to maintain sample integrity. Repeated freeze-thaw cycles were avoided by preparing separate aliquots before storage. Standardized sample handling and processing procedures were followed to minimize pre-analytical variability that could affect biomarker measurements.

Biochemical analysis

Serum VEGF and PlGF concentrations were measured using commercially available human VEGF and human PlGF enzyme-linked immunosorbent assay (ELISA) kits according to the manufacturer’s instructions. The assays were based on the sandwich ELISA principle, using pre-coated microplate wells, specific enzyme-linked detection antibodies, substrate reaction, and optical density measurement for quantitative estimation of serum VEGF and PlGF concentrations. Calibration standards and quality-control samples supplied with the kits were used for each assay run. The kit manufacturer, catalog number, detection range, analytical sensitivity, intra-assay coefficient of variation, and inter-assay coefficient of variation were recorded from the respective kit inserts before analysis and were followed according to the manufacturer’s specifications.

The results of all assays were obtained under controlled laboratory conditions to ensure accuracy and reproducibility. As universally accepted pregnancy-specific reference ranges for serum VEGF and PlGF in early gestation are not well established, the diagnostic cut-off values in the present study were derived from receiver operating characteristic (ROC) curve analysis. The optimal cut-off value was ≤682.6 pg/mL for VEGF and ≤73.6 pg/mL for PlGF for differentiating early pregnancy loss cases from healthy pregnant controls. The ELISA method was selected because of its sensitivity and specificity for quantitative estimation of circulating angiogenic factors. Subsequent statistical comparison of cases and controls was performed using the quantitative measurements obtained.

Statistical analysis

Data analysis was performed using IBM Corp. Released 2014. IBM SPSS Statistics for Windows, Version 20. Continuous variables were expressed as mean ± standard deviation, whereas categorical variables were expressed as frequencies and percentages. The independent Student’s t-test and chi-square test were used to compare cases and controls, as appropriate. Receiver operating characteristic (ROC) curve analysis was performed to assess the diagnostic performance of serum VEGF and PlGF, including area under the curve, sensitivity, specificity, and optimal cut-off values. Diagnostic indices were reported with 95% confidence intervals, wherever available, including the AUC and ROC-derived parameters. A p-value of less than 0.05 was considered statistically significant.

## Results

Baseline characteristics

The study involved 60 pregnant women, of whom 30 were diagnosed with early pregnancy loss, and 30 were healthy pregnant controls, all at less than 20 weeks of gestation. The demographic characteristics of the two groups were comparable. There was no statistically significant difference between cases and controls with respect to maternal age, indicating minimal age-related demographic bias. Similarly, gestational age did not differ significantly between the groups, suggesting that gestational-age-related physiological variation was unlikely to influence the comparative biomarker analysis. The absence of significant differences in these baseline parameters supports appropriate group comparability and strengthens the validity of subsequent biochemical comparisons. Table 1 summarizes the baseline characteristics of the study population.

**Table 1:** Baseline characteristics of study participants according to age and gestational age Data are expressed as mean ± standard deviation (SD). Each group included 30 participants, representing 50% of the total study population (N=60). p-values were calculated using the independent Student’s t-test. A p-value <0.05 was considered statistically significant, whereas p>0.05 was considered not statistically significant. All participants were pregnant women at less than 20 weeks of gestation.

	Controls, n=30 (50%)	Cases, n=30 (50%)	t value	p-value
Age (years)	24.4 ± 2.38	24.6 ± 2.61	-0.307	0.485
Gestational age (weeks)	12.63 ± 1.51	12.23 ± 1.67	-0.926	0.435

Serum VEGF levels

A significant decline in serum VEGF levels was observed in women with early pregnancy loss when compared to healthy pregnant controls. The reduced levels indicate impaired angiogenic activity, which is required for proper placental vascular development. The difference in serum VEGF levels between cases and controls was statistically significant, suggesting an association between reduced VEGF levels and early pregnancy loss.

Serum PlGF levels

Serum PlGF levels were significantly reduced in cases compared to controls, indicating altered placental angiogenic activity in women with early pregnancy loss. PlGF is important in trophoblast function and placental vascular remodeling, and its marked reduction in the present study supports its association with impaired placental angiogenesis. These findings suggest that PlGF may have diagnostic relevance in differentiating early pregnancy loss cases from healthy pregnant controls in this case-control setting. Table 2 shows the detailed comparison of both parameters between the groups.

**Table 2:** Comparison of serum VEGF and PlGF levels between early pregnancy loss cases and healthy pregnant controls Data are expressed as mean ± standard deviation (SD). Each group included 30 participants, representing 50% of the total study population (N=60). p-values were calculated using the independent Student’s t-test. A p-value <0.05 was considered statistically significant; p<0.001 was considered highly statistically significant. VEGF: Vascular Endothelial Growth Factor, PlGF: Placental Growth Factor

Test	Cases, n=30 (50%)	Controls, n=30 (50%)	t value	p-value
VEGF (pg/mL)	564.9 ± 380.1	1194.6 ± 539.4	5.22	<0.001
PlGF (pg/mL)	19.63 ± 14.7	293.04 ± 206.6	6.90	<0.001

Diagnostic performance of VEGF (ROC analysis)

Analysis of receiver operating characteristic (ROC) curves indicated that serum VEGF had good discriminatory ability for differentiating early pregnancy loss cases from healthy pregnant controls. The area under the curve indicated good diagnostic accuracy, with moderate sensitivity and specificity. The ROC-derived cut-off value supports the potential diagnostic use of VEGF as an adjunctive marker in this case-control population. VEGF ROC parameters are shown in Table 3.

**Table 3:** Diagnostic performance of VEGF based on ROC analysis The diagnostic performance of serum VEGF was evaluated using receiver operating characteristic (ROC) analysis. Diagnostic accuracy is represented by the area under the curve (AUC), sensitivity, specificity, and optimal cut-off value.

Area under curve (AUC)	0.833
Sensitivity	80.0 %
Specificity	76.7 %
Best cut off	≤682.6 pg/mL

**Figure 1:**
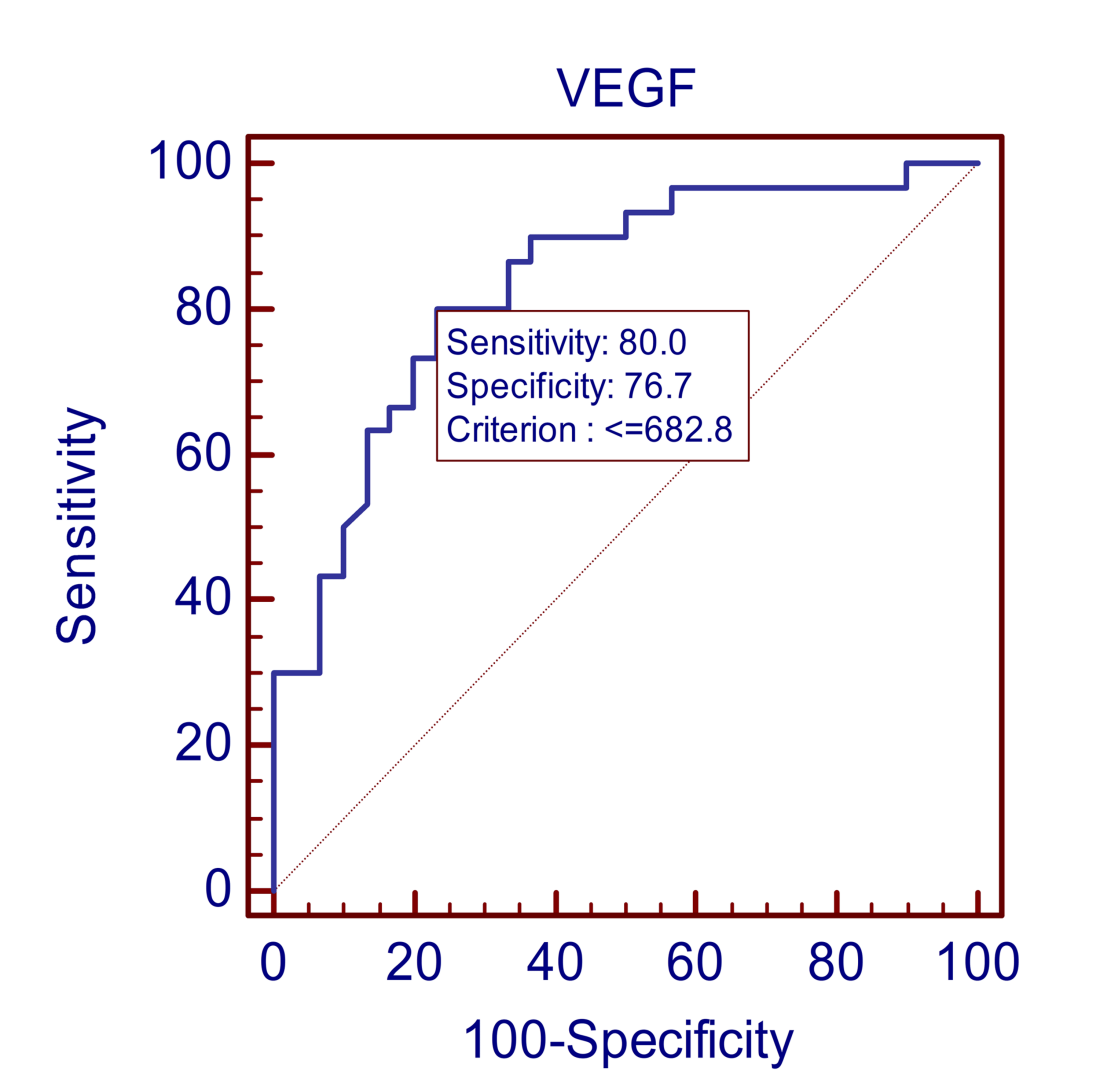
Receiver operating characteristic (ROC) curve for serum VEGF in the diagnosis of early pregnancy loss The ROC curve was generated to assess the diagnostic performance of serum VEGF in differentiating early pregnancy loss cases, n=30 (50%), from healthy pregnant controls, n=30 (50%). Diagnostic performance was expressed as the AUC, sensitivity, specificity, and optimal cut-off value. The AUC was 0.833, with a sensitivity of 80.0% and specificity of 76.7% at the optimal cut-off value of ≤682.6 pg/mL.

The corresponding ROC curve is illustrated in Figure 1.

Diagnostic performance of PlGF (ROC analysis)

Receiver operating characteristic (ROC) curve analysis showed that serum PlGF had excellent discriminatory ability for differentiating early pregnancy loss cases from healthy pregnant controls. The area under the curve indicated very high diagnostic accuracy in the present case-control sample, with optimal sensitivity and specificity at the derived cut-off value. These findings support the diagnostic performance of PlGF in this study population, while prospective validation is required before clinical predictive use can be inferred. The ROC parameters for PlGF are shown in Table 4.

**Table 4:** Diagnostic performance of PlGF based on ROC analysis The diagnostic performance of serum PlGF was evaluated using receiver operating characteristic (ROC) analysis. Diagnostic accuracy is represented by the area under the curve (AUC), sensitivity, specificity, and optimal cut-off value.

Area under curve (AUC)	1
Sensitivity	100%
Specificity	100%
Best cut off	≤73.6 pg/mL

**Figure 2:**
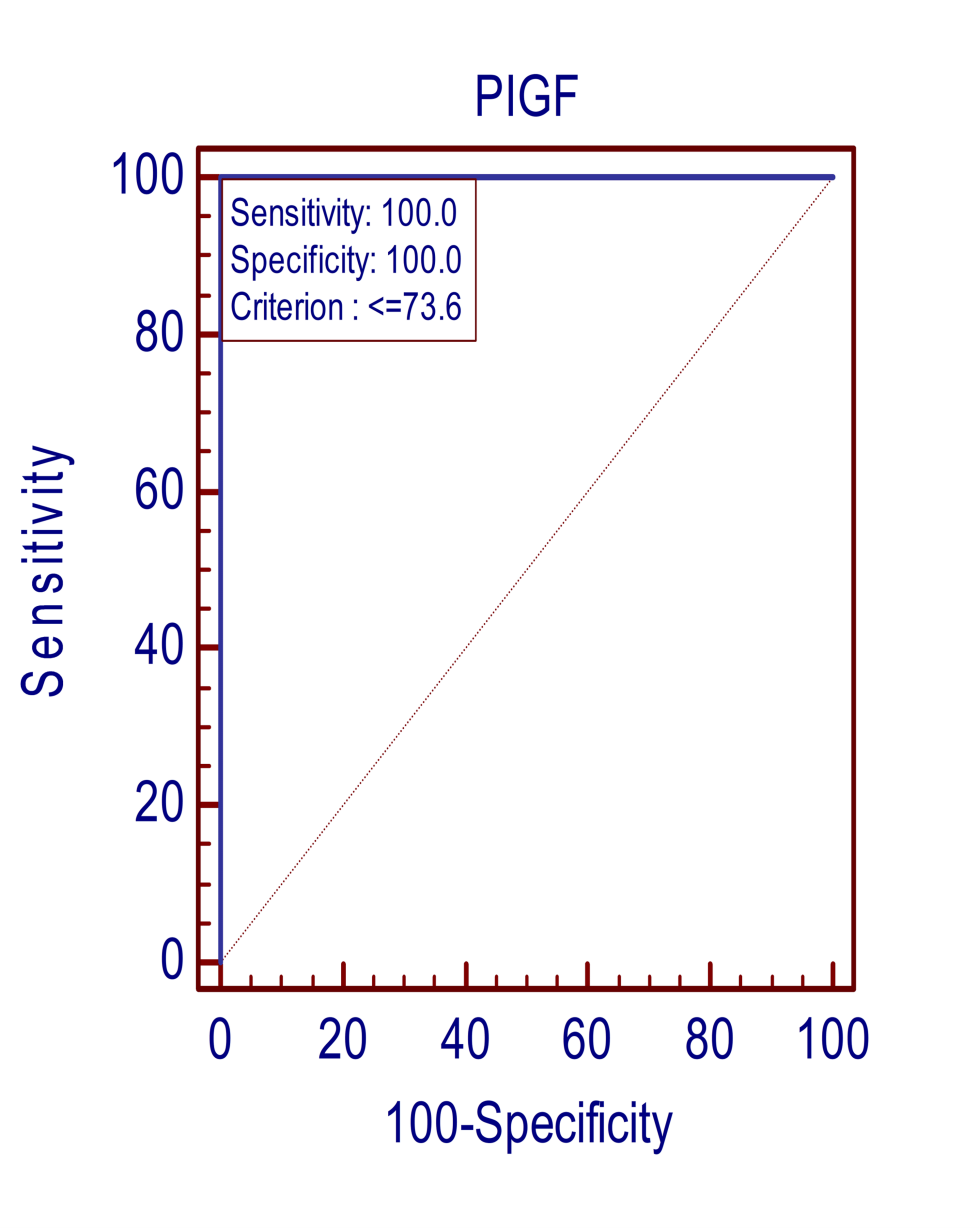
Receiver operating characteristic (ROC) curve for serum PlGF in the diagnosis of early pregnancy loss The ROC curve was generated to assess the diagnostic performance of serum PlGF in differentiating early pregnancy loss cases, n=30 (50%), from healthy pregnant controls, n=30 (50%). Diagnostic performance was expressed as the area under the curve (AUC), sensitivity, specificity, and optimal cut-off value. The AUC was 1.0, with a sensitivity of 100% and specificity of 100% at the optimal cut-off value of ≤73.6 pg/mL.

The corresponding ROC curve is illustrated in Figure 2.

Both VEGF and PlGF levels were markedly reduced in early pregnancy loss cases compared with healthy pregnancy controls. PlGF showed stronger diagnostic performance than VEGF in this case-control analysis, suggesting its potential role as an adjunctive diagnostic marker of altered placental angiogenesis in early pregnancy loss.

## Discussion

The current investigation shows a substantial decrease in serum VEGF and PlGF levels in women with early pregnancy loss compared with healthy pregnant controls. These results support the hypothesis that impaired angiogenesis may contribute to the pathophysiology of early pregnancy loss. The absence of significant differences in baseline characteristics, including age and gestational age, supports the validity of the observed biochemical differences and suggests that variation in angiogenic markers was less likely to be due to demographic or gestational-age-related confounding.

These findings are consistent with other studies that have reported reduced serum VEGF levels in women with recurrent pregnancy loss. Gupta et al. showed reduced VEGF levels in affected women compared with women with term pregnancies, highlighting the possible role of this factor in maintaining pregnancy [16]. Similarly, Amin et al. reported that VEGF gene sequence variations and altered expression were associated with recurrent pregnancy loss, supporting the biological relevance of VEGF signaling in adverse pregnancy outcomes [17]. VEGF polymorphisms have also been associated with recurrent miscarriage, indicating that both quantitative and qualitative disturbances in VEGF signaling may contribute to unfavorable pregnancy outcomes [18].

VEGF plays an important role in endothelial cell proliferation, migration, and vascular permeability, which are essential for placental development. Reduced VEGF levels may affect trophoblast invasion and vascular remodeling, resulting in poor placental perfusion and pregnancy loss. Research has indicated that angiogenic factors in early pregnancy are closely related to fetal growth and development and may influence pregnancy maintenance [19]. Experimental studies also support a relationship between VEGF expression, placental vascularization, and fetal development, suggesting that VEGF deficiency may impair oxygen and nutrient delivery to the developing embryo [20].

The current study also observed a considerable decrease in serum PlGF levels in cases of early pregnancy loss. PlGF, a placenta-associated angiogenic factor, plays an important role in trophoblast differentiation and vascular maturation. The marked reduction in PlGF observed in this study is consistent with previous reports. Zhang et al. found lower serum PlGF levels in cases of threatened abortion and premature delivery, supporting its relevance to pregnancy maintenance [21,22]. Likewise, Lian et al. emphasized the clinical relevance of PlGF in assessing recurrent spontaneous abortion and suggested that it may serve as a useful marker of adverse pregnancy outcomes [23].

Diagnostic performance analysis in the current study further supports the clinical relevance of these findings. VEGF exhibited good discriminatory performance, with an AUC of 0.833, sensitivity of 80.0%, and specificity of 76.7%, whereas PlGF showed excellent diagnostic performance, with an AUC of 1.0, sensitivity of 100%, and specificity of 100%. These findings suggest that PlGF had stronger diagnostic performance than VEGF in differentiating early pregnancy loss cases from controls in this case-control sample. The high diagnostic accuracy of PlGF may be related to its placental expression and direct involvement in placental vascular development, making it a potentially useful adjunctive diagnostic marker of placental dysfunction. However, because of the observational case-control design, these findings should not be interpreted as evidence of prospective predictive ability without validation in longitudinal studies. The very high diagnostic performance of PlGF, including an AUC of 1.0 with 100% sensitivity and specificity, should also be interpreted cautiously because it may reflect the small sample size, overfitting, or a case-control spectrum effect.

The interactions between angiogenic factors and immune mechanisms may also contribute to pregnancy maintenance. Altered expression of VEGF and its receptors has been demonstrated in trophoblastic tissue in recurrent pregnancy loss, often in association with immune dysfunction [21]. This suggests that impaired angiogenesis may be part of a broader pathological network involving inflammation, immune imbalance, and placental dysfunction.

Although the study has strengths, including clearly defined inclusion criteria, gestational-age comparability, and standardized biochemical assays, some limitations should be considered. The relatively small sample size may limit the generalizability of the findings. The hospital-based case-control design allows assessment of association and diagnostic discrimination but does not establish temporality or causality. Moreover, single-time-point biomarker measurement does not allow evaluation of dynamic changes in VEGF and PlGF levels across pregnancy. Pregnancy outcomes among controls were not followed longitudinally, and serial biomarker measurements were not performed. Important potential confounders, including body mass index, parity, prior miscarriage history, socioeconomic factors, smoking status, medication use, and inflammatory status, were not fully reported or adjusted for in the analysis. These limitations may affect external validity and should be addressed in future studies. These findings should be validated in larger prospective longitudinal studies to determine causal relationships, temporal biomarker patterns, and clinical applicability.

Overall, the results of this study provide additional evidence that angiogenic dysregulation is associated with early pregnancy loss. The marked reduction in serum VEGF and PlGF levels highlights their potential role as adjunctive diagnostic biomarkers of altered placental angiogenesis in affected women. Future prospective studies incorporating angiogenic marker assessment may help clarify their diagnostic utility, temporal behavior, and clinical relevance; however, routine screening or clinical implementation requires validation in larger longitudinal cohorts with standardized reference ranges.

## Conclusions

The present study demonstrated significantly reduced serum VEGF and PlGF levels in women with early pregnancy loss compared with healthy pregnant controls, suggesting an association between impaired angiogenesis and unfavorable early pregnancy outcomes. These findings support the importance of adequate placental vascular maturation and perfusion in maintaining early pregnancy. The reduction in these angiogenic markers indicates their possible involvement in the pathophysiology of unexplained early pregnancy loss, particularly when other known etiological factors are absent. The findings also suggest that VEGF and PlGF may have adjunctive diagnostic relevance for identifying altered angiogenic profiles in women with early pregnancy loss. However, because this was a hospital-based case-control study, the results should be interpreted as evidence of association and diagnostic discrimination rather than prospective prediction. Larger prospective longitudinal studies are needed to validate these findings, establish standardized reference values, and determine whether these biomarkers can be reliably incorporated into routine clinical practice.
